# Rhabdomyolysis as an initial presentation of systemic lupus erythematosus: a case report

**DOI:** 10.1186/s12245-019-0251-x

**Published:** 2019-11-08

**Authors:** Gayatri Saxena, Ahmed Mahdi

**Affiliations:** 1 St George’s Hospital, Blackshaw Road, Tooting, London, SW17 0QT UK; 2 Royal Surrey County Hospital, Egerton Road, Guildford, GU2 7XX UK

**Keywords:** Systemic lupus erythematosus, Rhabdomyolysis, Oral contraceptive, Myopathy

## Abstract

**Background:**

Systemic lupus erythematosus (SLE) is a multi-system autoimmune disease which most commonly presents in women of reproductive age. It takes a relapsing-remitting course and may manifest as a variety of clinical symptoms, making it difficult to diagnose at first presentation, particularly in the emergency department (ED) setting. In active SLE, rhabdomyolysis has, thus far, not been reported as the sole initial presentation.

**Case presentation:**

A 28-year-old presented to the ED with bilateral proximal arm swelling and pain. She had a previous history of Raynaud’s disease. Creatine kinase was > 13,000 units/l (normal range 25–200), but renal function was preserved. She did not require hospital admission so was encouraged to take oral fluids and discontinue the combined oral contraceptive pill. Antinuclear antibody and anti-double-stranded DNA titres were highly elevated with low complement demonstrated. She was diagnosed with SLE and treated with an oral prednisolone course. Antibody titres remained high 6 months later, provoking the initiation of hydroxychloroquine therapy.

**Conclusions:**

We report with a view to recommend autoimmune screening in young patients for whom a cause of rhabdomyolysis is not clearly identified.

## Background

Systemic lupus erythematosus (SLE) is a relapsing-remitting multi-system autoimmune disease with a female to male ratio of 9:1. The most common symptoms include arthralgia and photosensitive rash. It is usually diagnosed in the outpatient setting, based on clinical symptoms and biochemical markers. SLE may be life-threatening if there is significant cardiac, respiratory or renal involvement. Acute presentations of SLE to the emergency department are more readily identified in patients with a pre-existing diagnosis. However, due to significant heterogeneity in the symptoms of acute flares, rarer first time presentations are more likely to be overlooked, particularly in the context of emergency department time pressures.

## Case presentation

A 28-year-old Caucasian woman presented to the emergency department (ED) with a 24-h history of bilateral proximal arm swelling and pain following very mild exercise. Swelling and tenderness on palpation of these areas was noted, but clinical examination was otherwise unremarkable. She reported some self-limiting joint stiffness while abroad 1 month prior, which involved her wrists, elbows, knees and hips. Significant past medical history included Raynaud’s disease and recurrent urinary tract infections which resulted in mild renal scarring on ultrasound and associated chronic creatinine elevation. She was a lifelong non-smoker with infrequent alcohol consumption. She exercised regularly and took only the combined oral contraceptive pill. The patient had attended outpatient cardiology clinic 1 year before this ED attendance, complaining of a 5-month history of daily palpitations. Twelve-lead electrocardiography showed bigeminy due to frequent ventricular ectopics with a left bundle branch block morphology and inferior axis. No structural abnormalities were identified on transthoracic echocardiogram or cardiac magnetic resonance imaging. The ectopic beats were noted to diminish with exercise. Non-sustained ventricular tachycardia was detected on 24-h Holter monitoring. Bisoprolol was therefore commenced, and the patient was listed for electrophysiological studies with a view to undergo ablation for “paroxysmic benign arrhythmia”.

During the episode of bilateral arm swelling, vital signs were within normal limits: heart rate 75, blood pressure 108/73, respiratory rate 16 and oxygen saturations 100% on room air. Urinalysis showed trace protein with 1+ blood, but there was no obvious myoglobinuria. Serum creatine kinase (CK) was elevated at 13776, and creatinine was mildly raised at 106 mmol/l (chronic). There were no other remarkable blood test results at that time. A chest radiograph was normal. Exertional rhabdomyolysis was considered likely, so the patient was advised to discontinue her combined oral contraceptive pill and encouraged to take oral fluids. Doppler ultrasound of both upper extremities was negative for deep vein thrombosis. An autoimmune screen was later sent because the patient’s low intensity exercise prior to ED attendance was unlikely to have caused exertional rhabdomyolysis and there was no history of trauma.

The autoimmune screen revealed strongly positive antinuclear antibody (ANA), anti-Ro antibody, anti-La antibody, anti-ribonucleoprotein and anti-Sm antibody titres. Anti-double-stranded DNA antibody (anti-dsDNA) was also elevated with low complement levels. A borderline leukopenia was observed of 4.1, and erythrocyte sedimentation rate (ESR) was raised at 20. Ultrasound showed mild splenomegaly. The patient was deemed to have systemic lupus erythematosus, with an acute presentation of rhabdomyolysis.

Steroid treatment was initiated at 60 mg oral prednisolone and tapered by 10 mg every 2 weeks, followed by 5 mg for 2 weeks before cessation. Concomitant vitamin D and proton pump inhibitor were given, and a 3-L daily fluid intake was advised. CK improved but 6 months later, SLE disease activity remained significant according to biochemical markers such as anti-dsDNA, ESR and complement. Furthermore, the risk of disease progression was high given the significant number of positive autoantibodies. The patient’s rheumatologist therefore commenced hydroxychloroquine.

## Discussion and conclusions

In 95% of cases, patients with SLE exhibit musculoskeletal involvement. Indeed, musculoskeletal symptoms are the most common initial manifestation of the disease, particularly arthralgia and generalised muscle ache [1]. Inflammatory rhabdomyolysis is a rare first presentation. In our patient, this initial acute SLE flare may have been exacerbated by taking the combined oral contraceptive pill. There is a possible association between hormonal contraceptives and the development of SLE [2, 3]. However, another study has shown that although oral contraceptives may amplify the creatine kinase rise following intense exercise, they do not affect muscle propensity for damage [4]. In addition to trauma, hypoxia, drug, electrolyte and genetic causes of rhabdomyolysis, there are also cases reported that have been precipitated by viral illness [5]. This implies that a host of inflammatory processes may be implicated in the pathophysiology of rhabdomyolysis. Our patient normally undertakes a significantly more intense exercise regime which could suggest that active SLE may lower a person’s threshold for exertional muscle injury and breakdown.

The diagnosis of rhabdomyolysis is not always apparent; indeed, less than 10% of patients present with the classical triad of muscle pain, raised CK and dark urine [6]. For our patient, there was no evidence of myoglobinuria in the ED. This may be explained by the fact that myoglobin has a short half-life of only 2–4 h. Consequently, serum concentrations may return to normal within 6–8 h while CK persists for considerably longer [7]. Therefore, the absence of myoglobinuria should not exclude rhabdomyolysis.

In up to 10% of rhabdomyolysis cases, there may be an underlying myopathy [8]. This is difficult to confirm and usually requires a delayed muscle biopsy to be obtained. Inflammatory myopathies may also present with myalgia and elevated CK but were thought less plausible in this patient given the acute history of muscle pain and swelling which is more typical of rhabdomyolysis. There was speculation that rhabdomyolysis in this case may have been precipitated by an underlying myopathy‚ given the presence of positive anti-Ro and anti-La antibodies which have been associated with non-specific myositis [9]. However, the absence of chronicity, in the history of presenting complaint, made this less likely.

There are a few cases described in the literature which demonstrate an association between rhabdomyolysis and autoimmune disease [10, 11]. The relationship with SLE, in particular, is still not well-defined, though there are suggested links [12, 13]. There is no comment at this time on the sensitivity and specificity of rhabdomyolysis for SLE, so more studies are required to elicit this. Our case report aims to emphasise the importance of an autoimmune screen in young patients presenting with rhabdomyolysis because, in conjunction with immunological markers, the diagnosis may be established in a timely manner, prompting earlier treatment and better patient outcomes.

## Data Availability

Not applicable

## Abbreviations

ANA: Antinuclear antibody
CK: Creatine kinase
dsDNA: Double-stranded DNA
ED: Emergency department
ESR: Erythrocyte sedimentation rate
SLE: Systemic lupus erythematosus
